# Left vagus nerve magnetic stimulation facilitates nasogastric tube removal in post-stroke patients with dysphagia: a prospective observational cohort study

**DOI:** 10.3389/fneur.2026.1807489

**Published:** 2026-06-03

**Authors:** Shujuan Huang, Lirong Liu, Caixia Ouyang, Huimin Han, Yong Luo, Weifeng Wen, Si Chen, Hanbo Chen

**Affiliations:** Department of Rehabilitation Therapy, Guangdong Sanjiu Brain Hospital, Guangzhou, China

**Keywords:** dysphagia, nasogastric tube removal, prospective observational cohort study, stroke, vagus nerve magnetic stimulation

## Abstract

**Background:**

Post-stroke pharyngeal dysphagia (PSD) frequently leads to aspiration, aspiration pneumonia, and malnutrition, significantly compromising patient outcomes. Although standard swallowing rehabilitation training is widely applied, many patients show slow recovery with prolonged nasogastric tube placement. Recently, neuromodulation techniques such as vagus nerve magnetic stimulation (VNMS) have shown promise; however, observational evidence supporting its real-world clinical efficacy in post-stroke dysphagia patients remains limited.

**Methods:**

This single-blind prospective observational cohort study was conducted from June 2024 to June 2025 at Guangdong Sanjiu Brain Hospital, China. A total of 78 stroke patients with dysphagia were enrolled (33 in intervention group, 45 in control group). The intervention group received left-sided VNMS combined with standard swallowing rehabilitation training, while the control group received standard training alone. Multiple confounder adjustment and causal inference approaches were applied to ensure the robustness of the results.

**Results:**

Among 78 patients, after confounder adjustment, the intervention group demonstrated a significantly higher nasogastric tube removal rate, with an adjusted hazard ratio of 1.850 (95% CI: 1.103–3.101, *p* = 0.021). The intervention group removed nasogastric tubes on average 3.75 days earlier than the control group (95% CI: −5.01 to −2.50, *p* = 0.000), and the direction of effect was consistent across all analyses. Although swallowing safety assessment showed initial improvement, effects on overall swallowing function were inconsistent across analyses.

**Conclusion:**

In this prospective observational cohort, left-sided VNMS significantly accelerated nasogastric tube removal in post-stroke dysphagia patients, with an average advancement of 3.75 days. However, the improvement in swallowing physiological parameters was inconsistent, suggesting that VNMS’s effects may be primarily concentrated on functional outcomes. Long-term efficacy and optimal candidate populations require further investigation. Randomized controlled trials are recommended to verify causal relationships and confirm clinical utility.

## 1 Introduction

Stroke is one of the leading causes of death and disability globally. PSD, a common neurogenic complication of stroke, has a global incidence of approximately 46.6% (95% CI: 40.2–53.0%), further underscoring its high prevalence in the stroke population (1). The clinical consequences of PSD are substantial: first, PSD significantly increases the risk of aspiration, with an aspiration rate of approximately 34%, subsequently leading to aspiration pneumonia (2). These two complications not only directly increase patient mortality but also substantially prolong hospital length of stay (3). Second, PSD necessitates prolonged enteral nutritional support for patients, particularly nasogastric tube placement, which severely compromises quality of life and patient dignity (2, 4, 5). Third, from a health-economics perspective, treatment of PSD and management of its complications substantially increase the medical burden (6). Together, these factors establish PSD as a critical clinical problem requiring priority attention in post-stroke care.

The pharyngeal phase of swallowing is a key stage in the entire swallowing process, involving coordinated control of multiple structures including the soft palate, tongue base, larynx, and esophagus (7). As the core component of afferent and efferent pathways, the vagus nerve plays a crucial role in maintaining pharyngeal reflexes and coordinating swallowing, and therefore represents a potential therapeutic target (8–10). Current standard treatment for PSD primarily includes swallowing rehabilitation training, diet modification, speech therapy, and enteral nutrition support (11). Among adjunctive neurostimulation approaches, pharyngeal electrical stimulation (PES)—which delivers electrical stimulation to the pharyngeal mucosa via an intraluminal catheter electrode to activate afferent vagal pathways and promote cortical reorganization of swallowing networks—has been incorporated into current AHA stroke care guidelines as a treatment to be considered for dysphagia (Class IIb) (12–14). Nevertheless, the overall efficacy of currently available therapies remains limited, making it urgent to explore additional neuromodulatory interventions that may offer complementary or superior mechanisms of action (15–17).

In recent years, neuromodulation techniques—particularly VNMS—have attracted considerable attention in neurorehabilitation (18–20). VNMS is a noninvasive neuromodulatory technique that uses magnetic stimulation of the vagus nerve to promote neural plasticity and synaptic remodeling, thereby improving neural function (19). The vagus nerve, given its extensive neuroanatomical distribution—innervating pharyngeal muscles, the dorsal motor nucleus of the vagus, and related brainstem structures—plays a critical role in swallowing control (8, 21–22). Animal experiments and preliminary clinical studies suggest that VNMS combined with rehabilitation training can improve post-stroke swallowing and motor function (19, 23–24). Potential mechanisms include upregulation of Brain-Derived Neurotrophic Factor expression, promotion of cortical remodeling and neural plasticity (25); noninvasive vagus nerve stimulation via the cervical or auricular region (Peripheral vagus nerve stimulation/Transcutaneous auricular vagus nerve stimulation) may modulate inflammation and remodeling through vagal pathways, though applications in dysphagia remain limited (19, 26–28).

Existing literature on VNMS and swallowing function consists primarily of scattered case reports or small-scale clinical observations, lacking prospective, systematic cohort data. More importantly, these limited studies have not evaluated VNMS’s effects on major functional outcomes (such as time to nasogastric tube removal) in real-world clinical settings, nor have they analyzed characteristics of patient subgroups, making it impossible to provide evidence-based guidance for clinical decision-making and patient selection. To address this knowledge gap, we designed and conducted this prospective observational cohort study to evaluate the clinical effectiveness of left-sided VNMS compared with standard swallowing rehabilitation alone in patients with post-stroke dysphagia in a real-world clinical context. Our primary hypothesis was that left-sided VNMS combined with standard swallowing rehabilitation would significantly accelerate nasogastric tube removal compared with rehabilitation training alone, and reduce patients’ duration of dependence on enteral nutrition support. The primary study objective was to assess the causal effect of VNMS on time to nasogastric tube removal, defined as the number of days from treatment initiation to formal nasogastric tube removal, with follow-up for 28 days post-discharge, during which nasogastric tube removal was recorded as the primary time-to-event outcome. Secondary study objectives included: assessing the degree of VNMS-induced improvement across multiple swallowing function scales, including Standard Swallowing Assessment (SSA), Modified Water Swallow Test (MWST), Food Intake Level Scale (FILS), and Penetration-Aspiration Scale (PAS); evaluating VNMS’s effects on physiological outcomes, including vocal fold mobility improvement and tracheostomy decannulation rate; and using propensity score (PS)-stratified analysis to identify treatment effect heterogeneity across different patient subgroups, providing evidence for patient selection and individualized therapy in the era of precision medicine.

## 2 Methods

### 2.1 Study design and setting

This was a single-center, prospective, real-world observational cohort study conducted at Guangdong Sanjiu Brain Hospital in Guangzhou, China, from June 12, 2024, to June 11, 2025. The study adhered to the ethical principles of the Declaration of Helsinki and received approval from the Institutional Review Board of Guangdong Sanjiu Brain Hospital (approval number: 2024–01-034). All participants or their legal guardians provided written informed consent after full disclosure of study information. This study aimed to evaluate, in a real-world clinical context, the clinical effectiveness of left-sided V**NM**S compared with standard swallowing rehabilitation training in patients with post-stroke pharyngeal dysphagia, particularly its impact on nasogastric tube removal time, and to assess result robustness through multiple methods including PS, propensity score matching (PSM), and augmented inverse probability weighting (AIPW).

### 2.2 Study population

The study included patients meeting the following criteria: (1) acute or chronic stroke (ischemic or hemorrhagic) confirmed by neuroimaging (head CT or MRI): ischemic stroke manifesting as acute focal neurological deficit with CT/MRI showing ischemic changes, excluding hemorrhagic stroke and non-vascular etiologies; hemorrhagic stroke presenting with acute-onset focal or global neurological deficit, often accompanied by headache, vomiting, hypertension, and varying degrees of consciousness impairment, with CT/MRI showing hemorrhagic lesion, excluding ischemic stroke and non-vascular etiologies; (2) first-ever stroke event with pharyngeal dysphagia confirmed by flexible endoscopic evaluation of swallowing (FEES); (3) clinically stable status; (4) written informed consent signed by patient or legal guardian.

The study excluded patients with: (1) age < 18 years; (2) left mastoid skin damage, infection, or bone defect affecting coil placement; (3) dysphagia attributable to traumatic brain injury, intracranial neoplasm, or any non-vascular neurological etiology, irrespective of severity;(4) intracranial metallic implants, non-ferromagnetic drain tubes, or cardiac pacemakers; (5) prior transcranial ultrasound or peripheral magnetic stimulation; (6) history of epilepsy, family history of idiopathic epilepsy in first-degree relatives, or use of epileptogenic medications; (7) ear diseases affecting the left ear.

### 2.3 Enrollment and study flow

**Figure 1:**
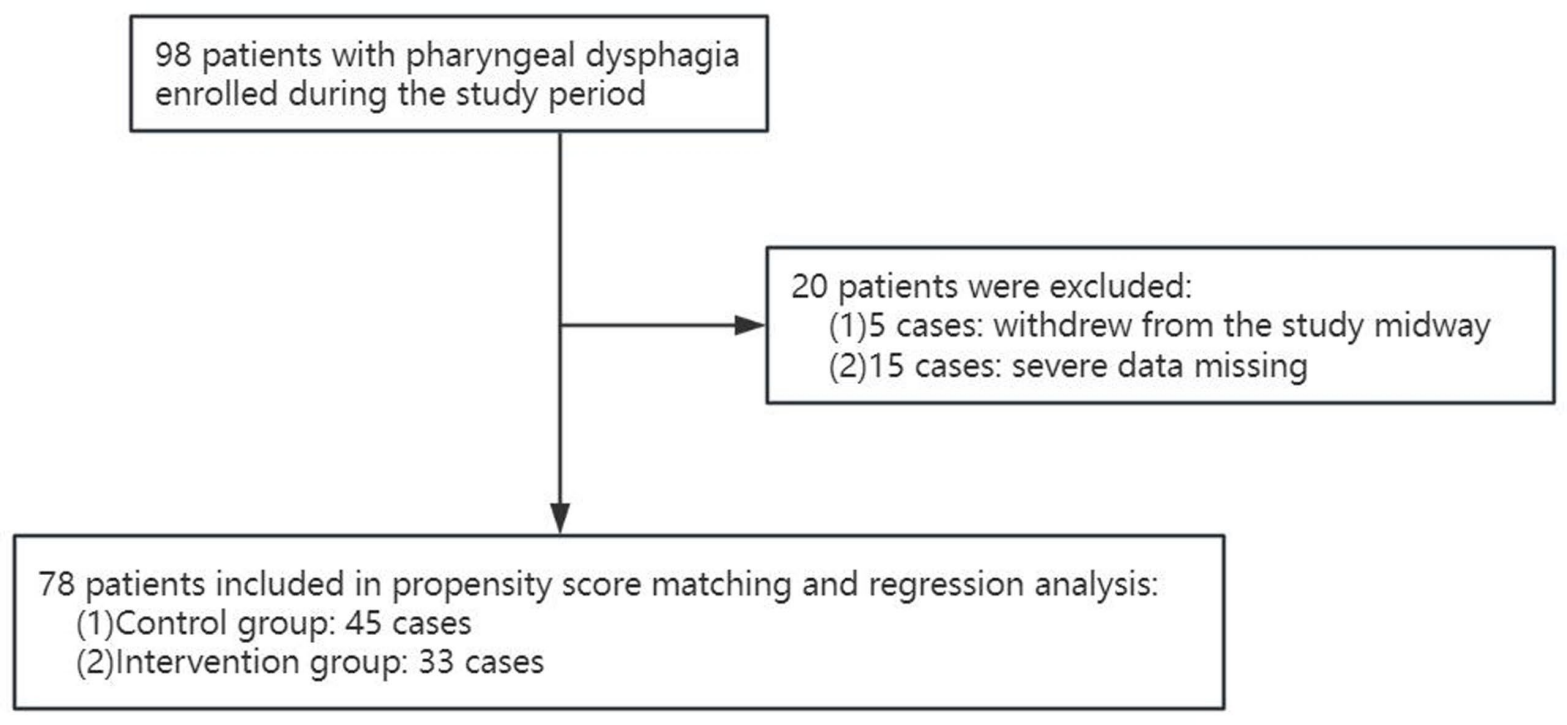
Study Cohort.

A total of 98 stroke patients (ischemic or hemorrhagic) with pharyngeal dysphagia were consecutively enrolled during the study period (June 12, 2024 – June 11, 2025). Twenty patients were subsequently excluded due to: (1) 5 patients who withdrew from the study midway; (2) 15 patients with severe data missing. This resulted in a final cohort of 78 patients (45 in the control group and 33 in the intervention group) who were included in the propensity score matching and regression analyses (see Figure 1 for the study enrollment flowchart).

### 2.4 Exposure definition and control group

**Figure 2:**
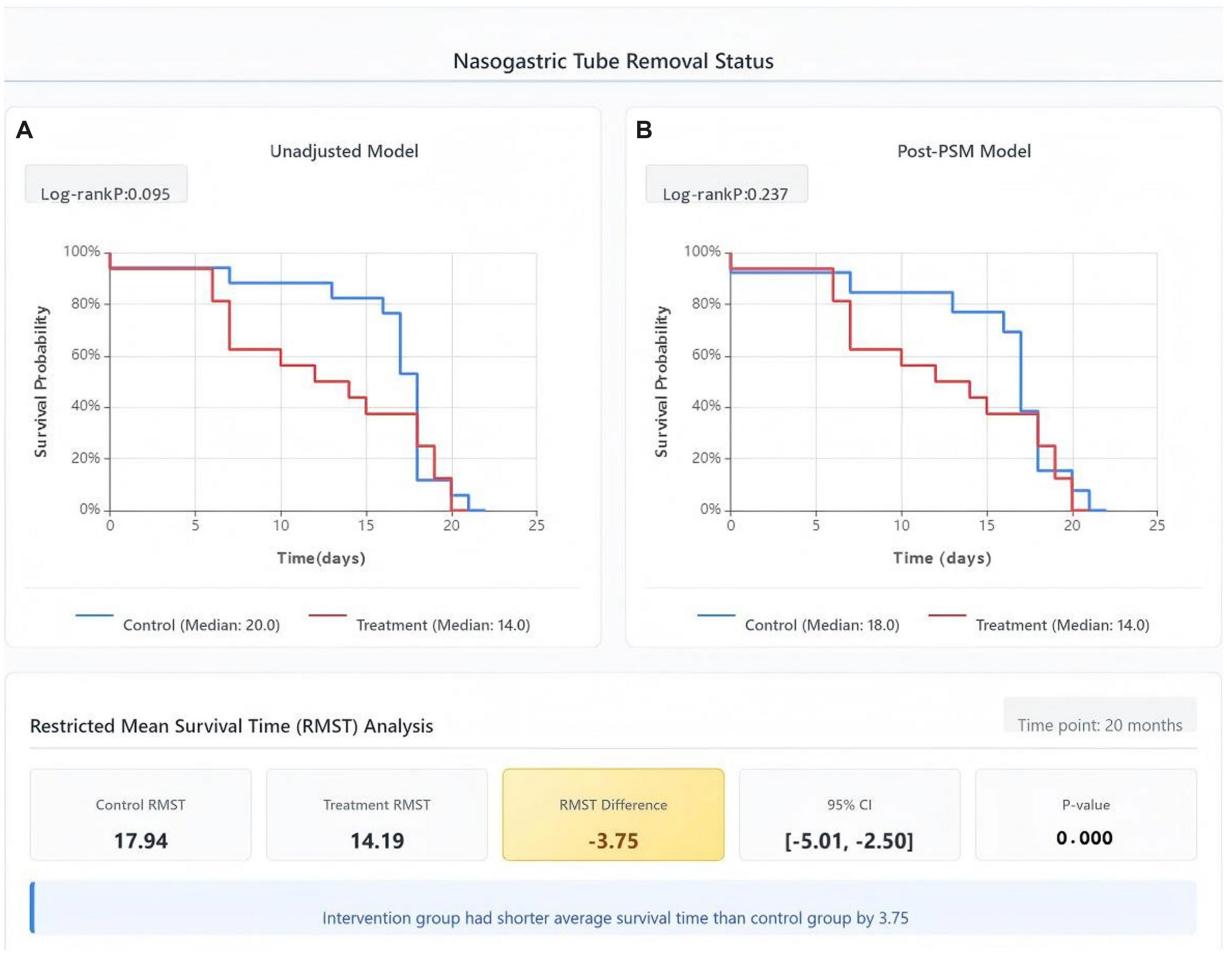
Survival analysis (primary outcome only) Kaplan-Meier curves and RMST analysis. **(A)** Unadjusted cohort. **(B)** Propensity score–matched cohort. The two subgraphs are generated from different analytical datasets (the original cohort and the matched cohort).

The intervention group received left-sided VNMS treatment. VNMS was delivered using a YDR CCY-I transcranial magnetic stimulation device (Yirder Company, Wuhan, China) equipped with a figure-eight-shaped coil (see Figure 2). To minimize potential cardiac complications (since the right vagus nerve traverses the atrioventricular node and may affect heart rate), the left vagus nerve was selected as the stimulation target. During treatment, patients were positioned in a lateral decubitus position, with the coil precisely placed at the left mastoid to stimulate the proximal segment of the vagus nerve at its exit from the jugular foramen. Stimulation parameters included pulse frequency of 5 Hz, intensity at 80% of the resting motor threshold, with a stimulation pattern of 6 s on followed by 24 s off, delivering 1,200 pulses per 20-min session (28).

The control group received standard swallowing rehabilitation training alone without VNMS treatment. Both groups underwent identical swallowing assessments and follow-up schedules over the 28-day post-discharge follow-up period. The control group assignment was based on shared decision-making among patients, families, and clinicians, reflecting real-world clinical practice.

Both groups received standard swallowing rehabilitation training, including motor training (Shaker exercises, tongue-strengthening exercises, pharyngeal muscle contraction exercises, etc.), sensory stimulation therapy (ice stimulation, electrical stimulation), oral intake and drinking training (progressively adjusting food consistency based on swallowing function grade), and patient education. Rehabilitation training was conducted 5 times per week for 30 min per session by trained speech-language pathologists.

### 2.5 Outcome definition and assessment

The primary outcome was time to nasogastric tube removal, defined as the time interval in days from treatment initiation to formal nasogastric tube removal. The criteria for nasogastric tube removal were: oral intake achievement with FILS ≥7 and absence of aspiration symptoms for 3 consecutive days. For patients with tracheostomy, oral feeding trials were initiated only after confirmation of: (1) adequate secretion management with reduced suctioning frequency; (2) successful cuff deflation tolerance; and (3) flexible endoscopic evaluation of swallowing (FEES) confirming pharyngeal swallowing safety with the cuff deflated. Full decannulation was not required as a prerequisite for FILS assessment, but cuff deflation assessment was mandatory prior to any oral intake trials in this subgroup. The follow-up period was 28 days post-discharge. Patients were followed from the date of discharge for a fixed period of 28 days, during which nasogastric tube removal status was monitored and recorded daily by the charge nurse or ward nurse in nursing records.

Secondary outcomes included: (1) changes in swallowing-related scales at baseline (1–3 days before treatment), 1–3 days after 3 weeks of treatment, including FILS, PAS, SSA, MWST, and Murray score;(2) physiological/clinical outcomes, including vocal fold mobility improvement and tracheostomy decannulation status. All swallowing assessments were performed by trained, blinded speech-language pathologists or specialists. Core outcomes such as FEES scores were completed by independent assessors blinded to group allocation.

### 2.6 Data sources

All patient data were extracted from the hospital electronic medical record system without manual extraction from paper charts. Collected data included: (1) demographic information: age, sex; (2) Stroke-related: stroke type, lesion location (supratentorial/infratentorial/mixed), stroke onset time; (3) baseline comorbidities: hypertension, diabetes, hyperlipidemia, pneumonia, etc.; (4) treatment-related: use of botulinum toxin, clonazepam, chlorpromazine, or other medications affecting saliva secretion; respiratory therapy, tracheostomy, and nasogastric tube placement details; (5) swallowing assessment tools and results: FEES, SSA, MWST, FILS, Murray, PAS scores; (6) intervention-related: actual number of VNMS sessions completed and parameters, adverse events; (7) rehabilitation training records: number and content of sessions completed; (8) follow-up data: complications, discharge outcomes.

### 2.7 Confounding and bias control

Based on clinical theory, prior published studies, and the epidemiological definition of confounding factors, the following variables that influence both receipt of VNMS and time to nasogastric tube removal were selected: age, stroke type, lesion location, baseline comorbidities, and relevant treatments. All variables were included in both the propensity score model and the outcome model to ensure the double robustness of the AIPW method.

To control potential confounding and selection bias, this study employed multi-level causal inference methods. First, propensity score was calculated during analysis to estimate patient probability of receiving VNMS treatment. Second, PSM using nearest-neighbor algorithm was performed with 1:1 matching and caliper width of 0.25 standard deviation; balance assessment was conducted post-matching with standardized difference <0.10. Third, AIPW, a doubly robust method, was employed by constructing propensity score and outcome models, calculating stabilized weights, reporting weight distribution, and implementing extreme weight handling strategy (weight truncation at the 99th percentile). The full results of covariate balance, including standardized mean differences (SMDs) and a love plot visualization, are provided in the Supplementary Tables S1, S2 and Supplementary Figure S1.

### 2.8 Statistical analysis

The primary analysis strategy centered on time-to-event data for nasogastric tube removal time, employing AIPW for causal estimation supplemented by five complementary analytical approaches to assess result robustness: (1) unadjusted Kaplan–Meier analysis; (2) multivariable Cox regression (adjusted for prespecified confounders); (3) propensity score-adjusted Cox model; (4) Kaplan–Meier analysis post-propensity score matching; (5) AIPW analysis (primary analysis). Proportional hazards assumption was tested for Cox models using Schoenfeld residuals; if *p* < 0.05 indicating assumption violation, stratified Cox model or interaction terms with time were employed.

Analysis of secondary outcomes included: longitudinal changes in swallowing function scales analyzed using linear mixed models, incorporating time point effect, group effect, and group-by-time interaction effect; categorical variables such as vocal fold mobility improvement and tracheostomy decannulation analyzed using logistic regression with OR and 95% CI. Missing data were handled using multiple imputation (MI), with 20 rounds of MI (m = 20) capturing uncertainty introduced by missingness, with results combined using Rubin’s rules. Sensitivity analyses assessed impact of missing data mechanisms (worst-case and best-case scenarios).

Multiple comparison correction: primary outcome used uncorrected significance threshold (*p* < 0.05); secondary outcomes used Bonferroni correction. Results presentation: time data presented as median and interquartile range (IQR), effect sizes as HR/OR with 95% CI, *p-*values precise to four decimal places. All statistical tests were two-sided with *α* = 0.05.

Subgroup analyses were conducted through propensity score stratification (quartiles) and clinically prespecified stratification, including stratification by age (≤65 years vs. > 65 years), stroke type, lesion location, and baseline swallowing function severity to assess treatment effect heterogeneity of VNMS. All statistical analyses were performed using R software (version ≥4.2.0) with packages including survival, WeightIt, cobalt, MatchIt, and mice.

## 3 Results

### 3.1 Baseline characteristics

Baseline characteristics of the two groups are shown in Table 1. The control group included 45 patients (32 male, 13 female; mean age 51.47 ± 14.33 years), while the intervention group included 33 patients (20 male, 13 female; mean age 49.39 ± 14.31 years). There were no significant differences between groups in gender distribution (χ^2^ = 0.945, *p* = 0.354) or age (t = 0.631, *p* = 0.529).

**Table 1:** Baseline characteristics.

Variable	Control group (*n* = 45)	Intervention group (*n* = 33)	Statistic	*P*-value
Gender				
Female	13 (28.9%)	13 (39.4%)	0.945	0.354
Male	32 (71.1%)	20 (60.6%)		
Age	51.47 ± 14.33	49.39 ± 14.31	0.631	0.529
Disease type				
Cerebral Hemorrhage	26 (57.8%)	21 (63.6%)	0.273	0.603
Cerebral Infarction	19 (42.2%)	12 (36.4%)		
Lesion location				
Supratentorial	24 (53.3%)	10 (30.3%)	6.531	0.038
Infratentorial	13 (28.9%)	19 (57.6%)		
Mixed	8 (17.8%)	4 (12.1%)		
Hypertension				
No	24 (53.3%)	17 (51.5%)	0.025	0.781
Yes	21 (46.7%)	16 (48.5%)		
Diabetes Mellitus				
No	40 (88.9%)	28 (84.8%)	N/A	0.735
Yes	5 (11.1%)	5 (15.2%)		
Hyperlipidemia				
No	30 (66.7%)	21 (63.6%)	0.077	0.728
Yes	15 (33.3%)	12 (36.4%)		
Pneumonia				
No	10 (22.2%)	5 (15.2%)	0.613	0.457
Yes	35 (77.8%)	28 (84.8%)		
Other complications				
No	23 (51.1%)	16 (48.5%)	0.053	0.750
Yes	22 (48.9%)	17 (51.5%)		
Disease Duration	61.00 (32.00–174.00)	82.00 (31.00–160.00)	0.634	0.527
Swallowing maneuvers				
No	1 (2.2%)	1 (3.0%)	N/A	1.000
Yes	44 (97.8%)	32 (97.0%)		
Balloon dilation				
No	34 (75.6%)	26 (78.8%)	0.112	0.701
Yes	11 (24.4%)	7 (21.2%)		
DC induction				
No	38 (84.4%)	28 (84.8%)	0.002	0.824
Yes	7 (15.6%)	5 (15.2%)		
Pulmonary rehabilitation techniques				
No	15 (33.3%)	9 (27.3%)	0.328	0.575
Yes	30 (66.7%)	24 (72.7%)		
Botulinum toxin				
No	45 (100.0%)	32 (97.0%)	N/A	0.423
Yes	0 (0.0%)	1 (3.0%)		
Amantadine				
No	31 (68.9%)	23 (69.7%)	0.006	0.814
Yes	14 (31.1%)	10 (30.3%)		
Clonazepam				
No	42 (93.3%)	29 (87.9%)	N/A	0.448
Yes	3 (6.7%)	4 (12.1%)		
Tracheostomy				
No	23 (51.1%)	18 (54.5%)	0.090	0.718
Yes	22 (48.9%)	15 (45.5%)		
Nasogastric tube placement				
No	2 (4.4%)	0 (0.0%)	N/A	0.506
Yes	43 (95.6%)	33 (100.0%)		

Continuous variables: Mean ± SD when mean > 2 × SD, otherwise Median (IQR); Categorical variables: n (%); Statistics: *t*-test shows *t-*value, U-test shows U value, Chi-square test shows *χ*^2^ value; Fisher exact test is automatically used for 2 × 2 tables when expected cell frequency < 5.

Disease characteristics were comparable between groups. The proportion of intracerebral hemorrhage was 57.8% in the control group and 63.6% in the intervention group (χ^2^ = 0.273, *p* = 0.603); the remainder were ischemic stroke. Disease duration from stroke onset to study enrollment did not differ significantly between groups (median 61.0 days, IQR 32.0–174.0 for the control group vs. 82.0 days, IQR 31.0–160.0 for the intervention group; *p* = 0.527), indicating that a substantial proportion of patients had been living with post-stroke dysphagia for weeks to months prior to entering the study.

However, lesion location distribution differed significantly (χ^2^ = 6.531, *p* = 0.038). The control group had a higher proportion of supratentorial lesions (53.3% vs. 30.3%), while the intervention group had a higher proportion of infratentorial lesions (57.6% vs. 28.9%).

Regarding comorbidities, hypertension prevalence was 46.7% in the control group and 48.5% in the intervention group (χ^2^ = 0.025, *p* = 0.781). Diabetes occurred in 11.1% vs. 15.2% (*p* = 0.735). Hyperlipidemia was present in 33.3% vs. 36.4% (χ^2^ = 0.077, *p* = 0.728). Pneumonia was the most common complication, affecting 77.8% of controls and 84.8% of the intervention group (χ^2^ = 0.613, *p* = 0.457). Other complications occurred in 48.9% vs. 51.5% (χ^2^ = 0.053, *p* = 0.750).

Treatment modalities and drug use were broadly comparable between groups. Nasogastric tube placement was used in 95.6% vs. 100% (*p* = 0.506). Tracheostomy was performed in 48.9% vs. 45.5% (χ^2^ = 0.090, *p* = 0.718). Swallowing rehabilitation was provided to 97.8% vs. 97.0% (*p* = 1.000). Balloon dilation was used in 24.4% vs. 21.2% (χ^2^ = 0.112, *p* = 0.701). Direct-current stimulation was applied to 15.6% vs. 15.2% (χ^2^ = 0.002, *p* = 0.824). Pulmonary rehabilitation techniques were used in 66.7% vs. 72.7% (χ^2^ = 0.328, *p* = 0.575). Amantadine was used in 31.1% vs. 30.3% (*p* = 0.814) and clonazepam in 6.7% vs. 12.1% (*p* = 0.448). No significant differences were observed.

Overall, both groups were well balanced at baseline, but lesion location differed significantly, which should be considered when interpreting subsequent results.

### 3.2 Primary and secondary outcomes

Five statistical methods were employed to analyze primary and secondary outcomes to assess result robustness, including: unadjusted analysis, multivariable adjustment, PS adjustment, PSM, and AIPW.

#### 3.2.1 Primary outcome: nasogastric tube removal status

In AIPW analysis, considered the most robust method, the intervention group demonstrated significantly higher nasogastric tube removal rate compared to the control group. The adjusted hazard ratio (HR) was 1.850 [95% CI: 1.103–3.101], *p* = 0.021, indicating that the hazard ratio for nasogastric tube removal in the intervention group was 1.85 times that of the control group. Although unadjusted analysis (HR = 1.645 [0.817–3.314], *p* = 0.163), multivariable-adjusted analysis (HR = 1.000 [0.039–25.381], *p* = 0.999), PS-adjusted analysis (HR = 1.892 [0.870–4.115], *p* = 0.108), and PSM analysis (HR = 1.811 [0.640–5.124], *p* = 0.263) did not reach statistical significance, the consistency in direction of effect estimates across these methods supported the credibility of the primary finding. It should be noted that the Kaplan–Meier median survival times represent the time point at which 50% of patients achieved nasogastric tube removal, rather than the overall completion rate within the follow-up window. The overall nasogastric tube removal rate within the 28-day post-discharge follow-up period was 53.8% (42 out of 78 patients). The remaining 46.2% (36/78) did not achieve nasogastric tube removal by the end of follow-up and were treated as right-censored observations in all time-to-event analyses. It should be emphasized that the Kaplan–Meier median survival times (20.0 days for the control group and 14.0 days for the intervention group) represent the time at which 50% of patients achieved removal, and do not imply that all patients achieved removal within the follow-up window. Among tracheostomized patients (37 patients total), 15 out of 37 (40.5%) achieved nasogastric tube removal within 28 days, compared with 27 out of 41 (65.9%) among non-tracheostomized patients, consistent with the greater clinical complexity of the tracheostomized subgroup.

#### 3.2.2 Secondary outcomes

Tracheostomy Removal Status: No significant differences were observed between groups across all five analytical methods (OR range: 0.79–4.06, all *p* > 0.05), suggesting that the intervention had no significant effect on tracheostomy removal rate.

SSA Improvement: In unadjusted analysis (*β* = 2.19 [0.34–4.05], *p* = 0.023) and multivariable-adjusted analysis (β = 2.15 [0.07–4.23], *p* = 0.044), the intervention group demonstrated statistically significant SSA score improvement, with improvement magnitude approximately 2–2.2 points. However, in PSM (*β* = −1.37 [−4.17–1.44], *p* = 0.339) and AIPW analysis (*β* = −3.73 [−24.78–17.32], *p* = 0.728), this effect attenuated, suggesting possible residual confounding after baseline adjustment.

Vocal Fold Mobility Changes: Notably, vocal fold mobility in the intervention group showed significant improvement in unadjusted analysis (*β* = 0.30 [0.03–0.57], *p* = 0.031), multivariable-adjusted analysis (β = 0.32 [−0.00–0.65], *p* = 0.050), and PS-adjusted analysis (β = 0.35 [0.04–0.65], *p* = 0.027). However, a reverse effect was observed in PSM analysis (β = −0.53 [−0.84−−0.21], *p* = 0.002), where the control group showed significantly greater vocal fold mobility improvement. This reverse effect indicates substantial baseline imbalance in vocal fold function between groups; these imbalances were not adequately captured in initial covariate adjustment, emphasizing the importance of sensitivity analysis in causal inference.

MWST, FILS, Murray Score for oropharyngeal secretions accumulation, Yale Pharyngeal Residue Severity Rating Scale, and the PAS: These swallowing-related metrics showed no significant differences between the two groups across all analyses (all *p*-values > 0.05), indicating that the intervention had no significant effect on these swallowing-related outcomes.

### 3.3 Propensity score stratification analysis

To examine whether intervention effects differed across different patient subgroups, we conducted propensity score quintile stratification analysis. Patients were stratified into five groups (Q1–Q5) based on baseline propensity score, and treatment effect was estimated separately within each stratum. *p*-values for interaction were used to test consistency of treatment effects across strata.

#### 3.3.1 Primary outcome: nasogastric tube removal status

The hazard ratio for nasogastric tube removal varied across different propensity score strata, ranging from 0.75 to 3.00. Although individual strata did not reach statistical significance, the interaction test was statistically significant (*p* = 0.032), indicating substantial heterogeneity in intervention effectiveness across different patient subgroups. The strongest treatment effect appeared in the third quintile (HR = 3.00 [95% CI: 0.55–16.38], *p* = 0.205), while the weakest effect appeared in the fourth quintile (HR = 0.75 [95% CI: 0.20–2.79], *p* = 0.668). This pattern suggests that the effectiveness of the intervention in promoting nasogastric tube removal depends on baseline patient characteristics captured by the propensity score.

#### 3.3.2 Secondary outcomes

Tracheostomy Removal Status: The intervention demonstrated consistent effects on tracheostomy removal across all propensity score strata (OR range: 1.00–2.00, all *p* > 0.05), with no significant interaction effect (*p* = 0.399), indicating that baseline characteristics did not lead to treatment effect heterogeneity.

SSA Improvement: The improvement magnitude of SSA across strata ranged from −1.27 to 4.67 points overall, with the second stratum approaching significance (*β* = 4.67 [95% CI: −0.11-9.44], *p* = 0.056). The interaction test revealed borderline significant heterogeneity (*p* = 0.116), suggesting possible treatment effect variation by propensity score stratification.

MWST Change: The intervention’s effect on MWST varied across strata (β range: −0.85 to 0.83), with a statistically significant interaction test (*p* = 0.027), indicating treatment effect heterogeneity. The *β* value in the third stratum was 0.83, approaching statistical significance (95% CI: −0.05-1.72, *p* = 0.064), suggesting potential treatment benefit in this patient subgroup.

FILS Change: A significant treatment-stratum interaction was detected (*p* = 0.030), with evident treatment effect heterogeneity across baseline propensity score categories. Notably, the third stratum demonstrated significant FILS score improvement in the intervention group (β = −2.44 [95% CI: −4.43 to −0.45], *p* = 0.018), indicating substantial improvement in swallowing function grade within this patient subgroup. This suggests that patients within the moderate propensity score range (third stratum) may derive the maximum functional benefit from the intervention.

Oropharyngeal Secretion Accumulation Scale (Murray Score) Change: The most pronounced heterogeneity was observed in Murray score changes, with highly significant interaction test (*p* = 0.006). The third stratum demonstrated statistically significant improvement (*β* = 1.17 [95% CI: 0.06–2.27], *p* = 0.041), indicating that the intervention group showed markedly superior management of oropharyngeal secretion accumulation in this patient subgroup compared to controls. Other strata (Q1, Q2, Q4, Q5) showed no significant differences. This significant interaction effect suggests that the intervention’s benefits for oropharyngeal secretion management are concentrated in a specific patient population characterized by moderate baseline propensity scores.

Vocal Cord Movement Change: The intervention’s treatment effect on vocal cord movement was relatively consistent across all propensity score strata (*β* range: −0.33 to 0.81), with no significant interaction detected (*p* = 0.285). This pattern indicates that the intervention produced no differential effects on vocal cord movement based on baseline characteristics.

Yale Pharyngeal Residue Severity Rating Scale Score Change: Although no individual propensity score stratum (Q1-Q5) demonstrated statistically significant improvement (*β* values ranged from −0.27 to 0.61, all *p* > 0.05), the interaction test was highly statistically significant (*p* = 0.005), indicating significant heterogeneity in treatment effects across all propensity score strata.

PAS Change: No significant interaction was detected in PAS changes (*p* = 1.000), indicating consistent treatment effects across all propensity score strata (although not statistically significant overall). Although the second stratum demonstrated significant improvement (β = 2.59 [95% CI: 0.53–4.65], *p* = 0.015), this appears to be an isolated finding with no consistent pattern observed in other strata.

### 3.4 Survival analysis of nasogastric tube removal status

We performed Kaplan–Meier survival analysis (including Log-rank test) and restricted mean survival time (RMST) analysis to evaluate nasogastric tube removal as a time-to-event outcome and compare differences between the intervention and control groups.

#### 3.4.1 Unadjusted model analysis

In the unadjusted model, the median survival time (i.e., time to nasogastric tube removal) was 20.0 days for the control group and 14.0 days for the intervention group. The Log-rank test did not reach statistical significance (*p* = 0.095), indicating that the difference in nasogastric tube removal time between groups was borderline non-significant in unadjusted analysis. RMST analysis at the 20-day time point revealed significant differences: RMST was 17.94 days for the control group and 14.19 days for the intervention group, with RMST difference of −3.75 days [95% CI: −5.01 to −2.50], *p* = 0.000. This indicates that patients in the intervention group removed nasogastric tubes on average approximately 3.75 days earlier than patients in the control group. See Figure 2A for details.

#### 3.4.2 Propensity score matching analysis

After PSM to reduce confounding bias, the matched cohorts demonstrated more comparable baseline characteristics. The median survival time for the control group remained at 18.0 days, while the median survival time for the intervention group remained at 14.0 days. The Log-rank test in PSM analysis was not statistically significant (*p* = 0.237), suggesting that propensity score matching attenuated the apparent effect of median survival time difference. Notably, RMST analysis at the 20-day time point continued to demonstrate statistically significant advantage in the intervention group, with RMST difference of −3.75 days [95% CI: −5.01 to −2.50], *p* = 0.000, indicating that the benefit of the intervention in accelerating nasogastric tube removal persists even after matching adjustment for confounding variables. Details are shown in Figure 2B.

## 4 Discussion

### 4.1 Summary of main findings

Through a prospective real-world observational cohort study, this study evaluated the clinical effectiveness of left-sided VNMS in 78 patients with post-stroke dysphagia. The most robust analytical method—AIPW analysis—demonstrated that the hazard ratio for nasogastric tube removal in the intervention group was 1.850 (95% CI: 1.103–3.101, *p* = 0.021), indicating that VNMS significantly accelerated nasogastric tube removal. All five analytical methods demonstrated consistent positive effects. RMST analysis further quantified this clinical benefit, with intervention group patients removing nasogastric tubes on average 3.75 days earlier (*p* = 0.000), representing meaningful progress in clinical practice.

However, no significant differences were observed in most secondary outcomes, including the MWST, FILS, and PAS. This discordance between the primary functional outcome and the secondary physiological parameters warrants careful interpretation. Several explanations are plausible: first, VNMS may preferentially reorganize the functional neural pathways governing the swallowing decision threshold (i.e., the transition from tube-dependent to oral feeding) without producing detectable changes in the specific physiological parameters captured by bedside swallowing scales within the 3-week treatment window; second, commonly used bedside instruments (SSA, MWST, FILS, PAS) may lack the sensitivity to detect subtle but functionally meaningful neurophysiological changes in a heterogeneous, high-acuity post-stroke population; third, Bonferroni correction for multiple comparisons substantially elevated the significance threshold for secondary outcomes, potentially obscuring modest but genuine improvements. Importantly, propensity score stratification analysis identified significant improvements in FILS and Murray scores within the third propensity score stratum (Q3), suggesting that improvements in physiological swallowing parameters may be concentrated in specific patient subgroups with intermediate baseline characteristics. These observations collectively do not negate the primary finding but highlight the importance of patient stratification and the need for future studies with longer follow-up and more sensitive physiological measurement tools.

### 4.2 Comparison with existing literature and heterogeneity findings

These results are consistent with the overall trend of prior vagus nerve stimulation studies. A recent systematic review and meta-analysis including 18 randomized controlled trials (954 participants) demonstrated that vagus nerve stimulation improved swallowing function in stroke patients (SMD = 0.62, *p* = 0.01) (29). Compared with our study, that meta-analysis encompassed multiple vagus nerve stimulation modalities, whereas our study specifically focused on noninvasive magnetic stimulation as a particular technique.

Notably, propensity score quintile stratification analysis revealed significant heterogeneity in treatment effects. The strongest treatment effect appeared in the third stratum (HR = 3.00), representing a population with intermediate propensity scores. Within this subgroup, improvements in both FILS and Murray scores reached statistical significance. This finding suggests that patients’ baseline characteristics (age, stroke type, lesion location, baseline swallowing function severity) are key determinants of treatment response. Patients with intermediate propensity scores may represent the population deriving maximum benefit from VNMS, providing important guidance for precision medicine applications and future patient selection.

### 4.3 Neurobiological mechanisms

Animal studies provide important insights into mechanisms by which vagus nerve stimulation improves swallowing function. In middle cerebral artery occlusion rat models, researchers observed beneficial changes at multiple levels following transcranial vagus nerve stimulation treatment: increased white matter myelination, increased capillary density, increased expression of vascular endothelial growth factor and basic fibroblast growth factor, and significantly suppressed expression of pro-inflammatory factors (IL-1β and TNF-*α*). These multiple mechanisms—demyelination repair, angiogenesis, and anti-inflammation—likely constitute the neurobiological foundation by which vagus nerve stimulation improves swallowing function (30).

Vagus nerve stimulation promotes neural plasticity and neural remodeling by activating key brainstem structures such as the nucleus tractus solitarius and locus coeruleus, facilitating release of norepinephrine, acetylcholine, and serotonin (31, 32). The pharyngeal phase of swallowing involves complex brainstem-medullary neural circuits, including coordination of the trigeminal, facial, glossopharyngeal, vagus, and hypoglossal nerves (7, 8, 9). VNMS improves swallowing function by enhancing functional integration of this complex neural circuit.

### 4.4 Clinical value and application significance

Nasogastric tube removal represents an objective, measurable milestone of swallowing function recovery. Prolonged nasogastric tube placement is associated with multiple complications, including increased aspiration risk, deteriorating oral hygiene, decreased quality of life, and psychological problems (33–36), and VNMS enables patients to remove nasogastric tubes on average 3.75 days earlier—reducing complications from prolonged artificial nutritional support, shortening hospital length of stay, and allowing earlier transition to oral nutrition; the downstream significance of this acceleration is further illuminated by Rouhi et al. (37), who conducted an entropy-balanced analysis comparing percutaneous endoscopic gastrostomy (PEG) and radiologically inserted gastrostomy (RIG) in 217 stroke patients with dysphagia and found that while both techniques achieved comparable time to goal feeds (3 vs. 4 days, *p* = 0.059), RIG conferred significantly lower perioperative morbidity including reoperation (AOR 0.10), cerebrovascular accident (AOR 0.24), and ICU admission (AOR 0.14), with mortality modulated by comorbidities such as arrhythmia, myocardial infarction, and obesity (37)—as these 217 patients all ultimately required permanent gastrostomy, they represent precisely the population that earlier nasogastric tube removal might help avoid, suggesting that VNMS may extend its clinical value beyond swallowing rehabilitation to the broader optimization of enteral nutrition management pathways by reducing progression to gastrostomy and its associated risks.

Furthermore, VNMS as a noninvasive neuromodulation technique offers unique advantages over surgically implanted vagus nerve stimulation devices by eliminating surgical risks (38–39), and over transcranial direct current stimulation by providing higher target precision via figure-eight coils, superior biosafety without current-related tissue damage, and better patient tolerance (40–41); notably, both our study and Rouhi et al. (37) converge on the importance of individualized patient selection—their finding that local referral patterns and comorbidity profiles influence gastrostomy outcomes parallels our propensity score stratification demonstrating that VNMS benefit is concentrated in patients with intermediate baseline characteristics, collectively reinforcing the need for precision-based patient selection in post-stroke dysphagia management.

### 4.5 Study strengths

This study employed a real-world observational cohort study design, enrolling a highly complex patient population (77.8–84.8% pneumonia, 45.5–48.9% tracheostomy), fully reflecting actual clinical practice. Second, this study employed five progressive statistical methods (unadjusted, multivariable-adjusted, propensity score-adjusted, propensity score matching, and AIPW) for analysis, with completely consistent effect direction of primary outcomes across all methods, substantially strengthening result robustness. Third, RMST analysis provided intuitive clinical interpretability, quantifying average benefit throughout the entire follow-up period. Fourth, propensity score stratification analysis identified the population with maximum benefit (third stratum), providing important information for precision medicine application. Fifth, stringent single-blinding design and quality control measures enhanced result credibility.

### 4.6 Study limitations

This study has the following major limitations: First, the observational design inherently limits causal inference. Although advanced statistical methods such as propensity score were employed, unmeasured confounders may still affect results. Second, the follow-up period of only 28 days post-discharge is relatively short; long-term effects of VNMS remain incompletely elucidated. Third, some secondary outcomes did not show significant improvement, which may suggest: (1) VNMS’s effects primarily concentrate on functional outcomes; (2) measurement of these physiological parameters may lack sufficient sensitivity. Finally, post-propensity score matching sample size was further reduced, affecting statistical power; lesion location differed significantly between groups (supratentorial lesion: control 53.3% vs. intervention 30.3%); although adjusted through propensity score, patients with different lesion locations may have different treatment responses.

## 5 Conclusion

Despite limitations of small sample size and observational design, this study provides the first real-world evidence supporting VNMS as a promising neuromodulation therapy for post-stroke dysphagia. Strong consistent evidence for the primary outcome (nasogastric tube removal), combined with identification of the population with maximum benefit (third stratum), establishes a foundation for clinical application and precision medicine strategies. For clinical practice, VNMS may serve as a supplementary treatment option for patients with limited benefit from conventional rehabilitation training, particularly for patient populations with intermediate propensity scores. However, large-scale randomized controlled trials are essential to confirm these findings.

## Data Availability

The datasets used and analyzed during the current study are available from the corresponding author on reasonable request.

## References

[1] Song W Wu M Wang H Pang R Zhu L. Prevalence, risk factors, and outcomes of dysphagia after stroke: a systematic review and meta-analysis. Front Neurol. (2024) 15:1403610. doi: 10.3389/fneur.2024.1403610, 39087010 PMC11288910

[2] Wang Z Shi R Moreira P. Post-stroke dysphagia: identifying the evidence missing. Front Med. (2025) 12:1494645. doi: 10.3389/fmed.2025.1494645 PMC11897572 40078394

[3] Banda KJ Chu H Kang XL Liu D Pien LC Jen HJ . Prevalence of dysphagia and risk of pneumonia and mortality in acute stroke patients: a meta-analysis. BMC Geriatrics. (2022) 22:420. doi: 10.1186/s12877-022-02960-5 35562660 PMC9103417

[4] Sobocki J Bogdanowska-Charkiewicz D Budnicka-Borkowicz A Chełmicka M Dudkowiak R Guzek M . Clinical Nutrition in Gastrointestinal Diseases: up to date Practice Guidance. Polish Soc Gastroenterol. (2025) 135:16967. doi: 10.20452/pamw.16967 40035369

[5] Volkert D Beck AM Cederholm T Cruz-Jentoft A Hooper L Kiesswetter E . ESPEN practical guideline: clinical nutrition and hydration in geriatrics. Clin Nutr. (2022) 41:91–6. doi: 10.1016/j.clnu.2021.11.011 35306388

[6] Marin S Serra-Prat M Ortega O Audouard Fericgla M Valls J Palomera E . Healthcare costs of post-stroke oropharyngeal dysphagia and its complications: malnutrition and respiratory infections. Eur J Neurol. (2021) 28:3670–81. doi: 10.1111/ene.14998 34176195

[7] Lang IM. Coordination of pharyngeal and esophageal phases of swallowing. J Neurogastroenterol Motility. (2024) 30:397–406. doi: 10.5056/jnm24003 PMC11474564 39397618

[8] Zainaee S Archer B Scherer R Bingman V Ghasemi M. Revealing goal-directed neural control of the pharyngeal phase of swallowing. Dysphagia. (2024) 40:528–40. doi: 10.1007/s00455-024-10758-3 39387924 PMC12145310

[9] Erman AB Kejner AE Hogikyan ND Feldman EL. Disorders of cranial nerves IX and X. Semin Neurol. (2002) 29:85–92. doi: 10.1055/s-0028-1124027, 19214937 PMC4239699

[10] Hasting S. The impact of person-based Factors on vagus nerve Stimulation Intensity and Responsiveness (Doctoral dissertation). Fort Worth, TX: Texas Christian University (2025).

[11] Dziewas R Michou E Trapl-Grundschober M Lal A Arsava EM Bath PM European Stroke Organisation and European Society for Swallowing Disorders guideline for the diagnosis and treatment of post-stroke dysphagia. Eur Stroke J. (2021). doi: 10.1016/j.esox.2021.100123 PMC8564153 34746431

[12] Bath PM Woodhouse LJ Suntrup-Krueger S Likar R Koestenberger M Warusevitane A . Pharyngeal electrical stimulation for neurogenic dysphagia following stroke, traumatic brain injury or other causes: Main results from the PHADER cohort study. EClinicalMedicine. (2020) 28:100608. doi: 10.1016/j.eclinm.2020.100608, 33294818 PMC7700977

[13] Dziewas R Stellato R van der Tweel I Walther E Werner CJ Braun T . Pharyngeal electrical stimulation for early decannulation in tracheotomised stroke patients with dysphagia (PHAST-TRAC): a randomised, single-blind, pivotal, superiority trial. Lancet Neurol. (2024) 17:849–59. doi: 10.1016/S1474-4422(18)30255-2 30170898

[14] Winstein CJ Stein J Arena R Bates B Cherney LR Cramer SC . Guidelines for adult stroke rehabilitation and recovery: a guideline for healthcare professionals from the American Heart Association/American Stroke Association. Stroke. (2016) 47:98. doi: 10.1161/STR.0000000000000098 27145936

[15] Chen X Ma L Hou M Gu X Wang Z Yao Y . Effects of rTMS on swallowing function and neuroimaging features in post-stroke dysphagia. Front Hum Neurosci. (2025) 19:1573083. doi: 10.3389/fnhum.2025.1573083 41416228 PMC12708527

[16] Xu F Zhang Y Su X Dai F Ye Y Ling M Effects of acupuncture combined with conventional rehabilitation training for patients with post-stroke dysphagia: a randomized controlled trial. J Multidiscip Healthc. (2025) 18:3139–52. doi: 10.2147/JMDH.S526827 40486255 PMC12143297

[17] Cakmak ET Sen EI Doruk C Sen C Sezikli S Yaliman A The effects of neuromuscular electrical stimulation on swallowing functions in post-stroke dysphagia: a randomized controlled trial. Dysphagia. (2023) 38:874–85. doi: 10.1007/s00455-022-10512-7 35986170

[18] Roy JM Musmar B Ritz C Sizdahkhani S Karadimas S Papadopoulos E Vagus nerve stimulation paired with rehabilitation for post-stroke recovery: a single center experience of patient satisfaction and outcomes. Clin Neurol Neurosurg. (2025) 257:109043. doi: 10.1016/j.clineuro.2025.109043 40633249

[19] Lin WS Chou CL Chang MH Chung YM Lin FG Tsai PY. Vagus nerve magnetic modulation facilitates dysphagia recovery in patients with stroke involving the brainstem: a proof of concept study. Brain Stimul. (2018) 11:264–70. doi: 10.1016/j.brs.2017.12.001 29162502

[20] Frasch MG Porges EC. Vagus nerve stimulation: state of the art of stimulation and recording strategies to address autonomic function neuromodulation. J Neural Eng. (2024) 13:041002. doi: 10.1088/1741-2560/13/4/041002 27351347

[21] Sun J Li X Zhou Y Liu H Ma C Hao C . Clinical advances in transcutaneous auricular vagus nerve stimulation for post-stroke disorders: state of the art and future perspectives. Front Neurol. (2025) 16:1676727. doi: 10.3389/fneur.2025.1676727 41245869 PMC12615202

[22] Ma L Wang HB Hashimoto K. The vagus nerve: an old but new player in brain–body communication. Brain Behav Immun. (2025) 124:28–39. doi: 10.1016/j.bbi.2024.11.023 39566667

[23] de Melo PS Parente J Rebello-Sanchez I Marduy A Gianlorenco AC Kim CK Understanding the neuroplastic effects of auricular vagus nerve stimulation in animal models of stroke: a systematic review and meta-analysis. Neurorehabil Neural Repair. (2023) 37:564–76. doi: 10.1177/15459683231177595 37272448

[24] Zhang Y Sun J Liu H. Transcutaneous vagus nerve magnetic stimulation: a bibliometric study on current research hotspots and status. Front Neurosci. (2024) 18:1406135. doi: 10.3389/fnins.2024.1406135, 39221007 PMC11363710

[25] Cai PY Bodhit A Derequito R Ansari S Abukhalil F Thenkabail S . Vagus nerve stimulation in ischemic stroke: old wine in a new bottle. Front Neurol. (2014) 5:107. doi: 10.3389/fneur.2014.00107, 25009531 PMC4067569

[26] Long L Zang Q Jia G Fan M Zhang L Qi Y . Transcutaneous auricular Vagus nerve stimulation promotes white matter repair and improves dysphagia symptoms in cerebral ischemia model rats. Front Behav Neurosci. (2022) 16:811419. doi: 10.3389/fnbeh.2022.811419, 35493949 PMC9051615

[27] Wang Y He Y Jiang L Chen X Zou F Yin Y . Effect of transcutaneous auricular vagus nerve stimulation on post-stroke dysphagia. J Neurol. (2022) 270:995–1003. doi: 10.1007/s00415-022-11465-5, 36329182

[28] Liu L Huang S Chen H Chen S Liang J Liu C. Vagal nerve magnetic stimulation for post-traumatic cricopharyngeal achalasia with bilateral vocal cord paralysis: a case report. Medicine. (2025) 104:e43525. doi: 10.1097/MD.0000000000043525, 40725869 PMC12303498

[29] Zhou J Sang M Shang X Gao X. Adjuvant effects of vagus nerve stimulation on post-stroke rehabilitation: a systematic review and meta-analysis. BMJ Open. (2025) 15:e106662. doi: 10.1136/bmjopen-2025-106662, 41469051 PMC12766840

[30] Long J Wang Y Liu J Liu H Sun Y Liu Z . Transcutaneous auricular vagus nerve stimulation promotes white matter remyelination and angiogenesis in MCAO rats. Neurosci Bull. (2023) 39:1055–68. doi: 10.1007/s12264-023-00985-5

[31] Wang L Gao F Dai Y Wang Z Liang F Wu J . Transcutaneous auricular vagus nerve stimulation on upper limb motor function with stroke: a functional near-infrared spectroscopy pilot study. Front Neurosci. (2023) 17:1297887. doi: 10.3389/fnins.2023.1297887 38075278 PMC10702495

[32] Yuan H Silberstein SD Bonaz B Sinniger V Pellissier S Badran BW . Mechanism and applications of Vagus nerve stimulation. Biomolecules. (2025) 15:122. doi: 10.3390/biom1502012239858516

[33] Huang S-T Chiou C-C Liu H-Y. Risk factors of aspiration pneumonia related to improper oral hygiene behavior in community dysphagia persons with nasogastric tube feeding. J Dental Scie. (2017) 12:375–81. doi: 10.1016/j.jds.2017.06.001, 30895078 PMC6395351

[34] Chen J Su Y Li T Mao X Jiang Q Yang Q . Aspiration risk prediction models in patients with nasogastric enteral nutrition: a systematic review and meta-analysis. Sci Rep. (2025) 15:26273. doi: 10.1038/s41598-025-12252-8, 40684043 PMC12276240

[35] Simpson AJ Allen J-L Chatwin M Crawford H Elverson J Ewan V . BTS clinical statement on aspiration pneumonia. Breathe. (2022) 18:210215. doi: 10.1183/20734735.0215 36863772

[36] Lisiecka D Kearns Á Evans W Farrell D. Aspiration pneumonia in nursing literature—a mapping review. J Clin Nurs. (2024) 32:4528–40. doi: 10.1111/jocn.16568 PMC11304538 39113687

[37] Rouhi AD Leon S Roberson JL Shreve LA Nadolski GJ Williams NN . Comparison of gastrostomy techniques in stroke patients with dysphagia: an entropy-balanced analysis. J Surg Res. (2024) 303:579–86. doi: 10.1016/j.jss.2024.09.064 39437597

[38] Madda DP Vempatapu S Kanaganuru S. Investigating the efficacy of non-invasive vagus nerve stimulation in treating drug-resistant epilepsy. Bioinformation. (2025) 6:973206300211551. doi: 10.1016/j.bioinfo.2025.06.030 PMC12449518 40978640

[39] Nonis R D'Ostilio K Schoenen J Magis D. Evidence of Activation of vagal Afferents by non-invasive vagus nerve Stimulation: An Electrophysiological Study in Healthy Volunteers. Liège: University of Liège (2025). 10.1177/0333102417717470 PMC5680905 28648089

[40] Gomez LJ Goetz SM Peterchev AV. Design of transcranial magnetic stimulation coils with optimal trade-off between depth, focality, and energy. J Neural Eng. (2018) 15:046033. doi: 10.1088/1741-2552/aac967 29855433 PMC6433395

[41] Bedder M Parker L. Magnetic peripheral nerve stimulation (mPNS) for chronic pain. J Pain Res. (2023) 16:2365–73. doi: 10.2147/JPR.S409331, 37465717 PMC10350402

