# Supplementary material for: Left vagus nerve magnetic stimulation facilitates nasogastric tube removal in post-stroke patients with dysphagia: a prospective observational cohort study
Source: Front Neurol. 2026 Jun 3;17:1807489. doi: 10.3389/fneur.2026.1807489 (PMC13271995; doi:10.3389/fneur.2026.1807489)
Supplement: Supplementary file 1 [file Table_1.docx]

**Table S1. Standardized Mean Differences (SMD) for Baseline Characteristics Before and After Propensity Score Matching**

| **Variable** | **Pre-Matching SMD** | **Post-Matching SMD** | **Balance Assessment** |
| --- | --- | --- | --- |
| Age | 0.126 | 0.045 | ✓ |
| Gender (Female) | 0.218 | 0.067 | ✓ |
| Disease Type (Hemorrhage) | 0.117 | 0.052 | ✓ |
| Lesion Location (Supratentorial) | 0.480 | 0.092 | ✓ |
| Lesion Location (Infratentorial) | 0.591* | 0.086 | ✓ |
| Hypertension | 0.035 | 0.018 | ✓ |
| Diabetes Mellitus | 0.118 | 0.056 | ✓ |
| Hyperlipidemia | 0.057 | 0.028 | ✓ |
| Pneumonia | 0.184 | 0.087 | ✓ |
| Other Complications | 0.049 | 0.024 | ✓ |
| Disease Duration | 0.142 | 0.061 | ✓ |
| Swallowing Maneuvers | 0.063 | 0.030 | ✓ |
| Balloon Dilation | 0.084 | 0.041 | ✓ |
| DC Induction | 0.011 | 0.005 | ✓ |
| Pulmonary Rehabilitation | 0.137 | 0.065 | ✓ |
| Botulinum Toxin | 0.187 | 0.089 | ✓ |
| Amantadine | 0.018 | 0.009 | ✓ |
| Clonazepam | 0.189 | 0.090 | ✓ |
| Tracheostomy | 0.069 | 0.033 | ✓ |
| Nasogastric Tube Placement | 0.315 | 0.095 | ✓ |

Note: SMD = Standardized Mean Difference. SMD < 0.10 indicates adequate covariate balance. Before PSM: n = 78 (Control 45, Intervention 33); After PSM: n = 60 (Control 30, Intervention 30 per group). *Lesion location (Infratentorial) showed the largest pre-matching imbalance (SMD = 0.591), which was effectively reduced to 0.086 after PSM. All baseline variables achieved SMD < 0.10 after matching, indicating adequate balance in the matched cohort.
