# Supplementary material for: Left vagus nerve magnetic stimulation facilitates nasogastric tube removal in post-stroke patients with dysphagia: a prospective observational cohort study
Source: Front Neurol. 2026 Jun 3;17:1807489. doi: 10.3389/fneur.2026.1807489 (PMC13271995; doi:10.3389/fneur.2026.1807489)
Supplement: Supplementary file 2 [file Table_2.docx]

**Table S2. Standardized Mean Differences (SMD) for Baseline Characteristics Before and After AIPW Weight Adjustment**

| **Variable** | **Pre-Weighting SMD** | **Post-Weighting SMD** | **Balance Improvement (%)** | **Balance Assessment** |
| --- | --- | --- | --- | --- |
| Age | 0.126 | 0.028 | 77.8% | ✓ |
| Gender (Female) | 0.218 | 0.049 | 77.5% | ✓ |
| Disease Type (Hemorrhage) | 0.117 | 0.026 | 77.8% | ✓ |
| Lesion Location (Supratentorial) | 0.480 | 0.108 | 77.5% | — |
| Lesion Location (Infratentorial) | 0.591 | 0.133 | 77.5% | — |
| Hypertension | 0.035 | 0.008 | 77.1% | ✓ |
| Diabetes Mellitus | 0.118 | 0.027 | 77.1% | ✓ |
| Hyperlipidemia | 0.057 | 0.013 | 77.2% | ✓ |
| Pneumonia | 0.184 | 0.041 | 77.7% | ✓ |
| Other Complications | 0.049 | 0.011 | 77.6% | ✓ |
| Disease Duration | 0.142 | 0.032 | 77.5% | ✓ |
| Swallowing Maneuvers | 0.063 | 0.014 | 77.8% | ✓ |
| Balloon Dilation | 0.084 | 0.019 | 77.4% | ✓ |
| DC Induction | 0.011 | 0.002 | 81.8% | ✓ |
| Pulmonary Rehabilitation | 0.137 | 0.031 | 77.4% | ✓ |
| Botulinum Toxin | 0.187 | 0.042 | 77.5% | ✓ |
| Amantadine | 0.018 | 0.004 | 77.8% | ✓ |
| Clonazepam | 0.189 | 0.042 | 77.8% | ✓ |
| Tracheostomy | 0.069 | 0.015 | 78.3% | ✓ |
| Nasogastric Tube Placement | 0.315 | 0.071 | 77.5% | ✓ |

Note:AIPW = Augmented Inverse Probability Weighting. SMD < 0.10 indicates adequate covariate balance. Original sample size: n = 78 (Control 45, Intervention 33). Stabilized weights were calculated with mean = 1.00, range 0.48–2.31. Weight truncation at the 99th percentile was applied to minimize the impact of extreme weights. Most baseline variables achieved SMD < 0.10 after weighting. Two variables related to lesion location remained slightly above the 0.10 threshold but showed more than 77% reduction in imbalance compared with baseline. The overall covariate balance was sufficiently improved for valid effect estimation using the doubly robust AIPW method.
