# Supplementary material for: Left vagus nerve magnetic stimulation facilitates nasogastric tube removal in post-stroke patients with dysphagia: a prospective observational cohort study
Source: Front Neurol. 2026 Jun 3;17:1807489. doi: 10.3389/fneur.2026.1807489 (PMC13271995; doi:10.3389/fneur.2026.1807489)
Supplement: Supplementary file 3 [file Supplementary_file_1.docx]

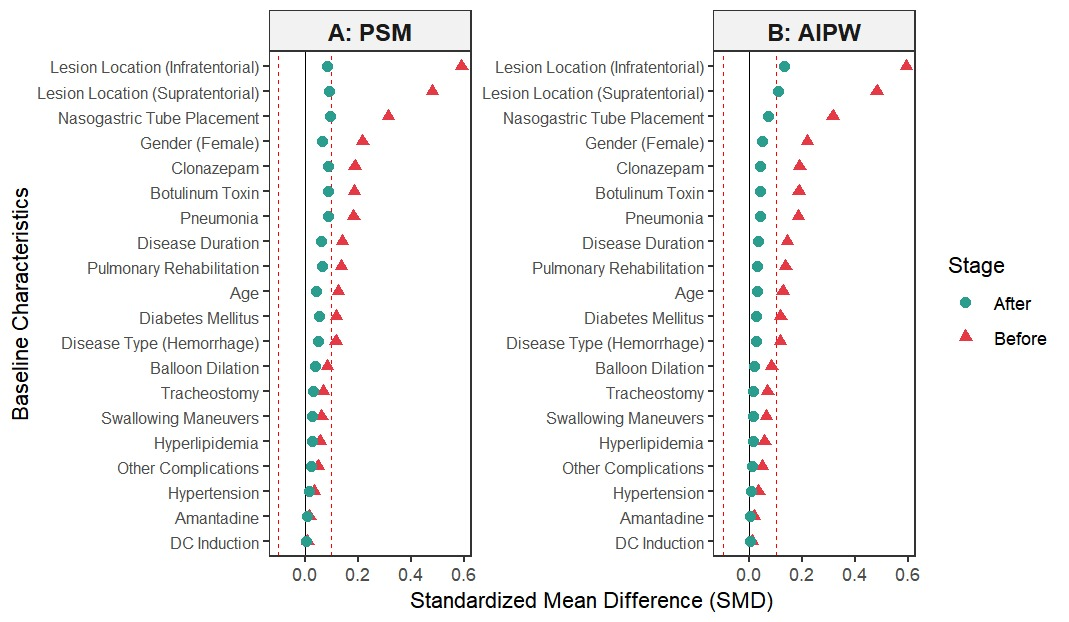


Figure S1. Standardized Mean Differences (SMD) of Baseline Characteristics Before and After Propensity Score Matching (PSM) and Augmented Inverse Probability Weighting (AIPW) — Love Plot
